# Selecting Core Outcomes for Randomised Effectiveness trials In Type 2 Diabetes (SCORE-IT): study protocol for the development of a core outcome set

**DOI:** 10.1186/s13063-018-2805-2

**Published:** 2018-08-07

**Authors:** Nicola L. Harman, John Wilding, Dave Curry, James Harris, Jennifer Logue, R. John Pemberton, Leigh Perreault, Gareth Thompson, Sean Tunis, Paula R. Williamson, on behalf of the SCORE-IT study team, Serena Battaglia, Jacques Demotes-Mainard, Valerie Gailus-Durner, Silvio Garattini, Cecilia A. C. Prinsen, Michael Raess, Patricia da Silva-Buttkus, Caroline B. Terwee

**Affiliations:** 1 Department of Biostatistics, Institute of Translational Medicine, University of Liverpool, Liverpool, L69 3GL UK; 2 Obesity and Endocrinology Clinical Research Group, Institute of Ageing and Chronic Disease, University Hospital Aintree, Longmoor Lane, Liverpool, L9 7AL UK; 3 Institute of Cardiovascular and Medical Sciences, University of Glasgow, Glasgow, UK; 4 Division of Endocrinology, Metabolism and Diabetes, Affiliate Center for Global Health, University of Colorado Anschutz Medical Campus, 13001 E. 17th Pl, Aurora, CO 80045 USA; 5 Colorado School of Public Health, 13001 E. 17th Place, Mail Stop B119, Aurora, CO 80045 USA; 6 Center for Medical Technology Policy (CMTP), World Trade Center Baltimore, Baltimore, MD USA

**Keywords:** Core outcome set, Systematic review, Type 2 diabetes

## Abstract

**Background:**

Type 2 diabetes is characterised by abnormal glucose metabolism, and treatment is aimed at normalising glycaemia. Outcomes measured in clinical trials should be meaningful to patients, health care professionals and researchers, yet there is heterogeneity in the outcomes used across trials of glucose-lowering interventions. This inconsistency affects the ability to compare findings and may mean that the results have little importance to health care professionals and the patients for whom they care. The SCORE-IT study aims to develop a core outcome set (COS) for use in all trials of glucose-lowering interventions for people with type 2 diabetes.

**Methods/design:**

This study will involve three key stages in the development of a COS: (1) A list of outcomes will be identified from multiple sources, specifically registered clinical trials, online patient resources, the qualitative literature and landmark studies identified by a Study Steering Committee. (2) The list of outcomes will be scored by multiple stakeholder groups in a two-round online international Delphi survey. (3) The results of the online Delphi will be summarised and discussed at a face-to-face consensus meeting with representation from all stakeholder groups.

**Discussion:**

The SCORE-IT study aims to develop an internationally relevant set of core outcomes for use in future trials of glucose-lowering interventions for type 2 diabetes. The use of a COS will improve the consistency of outcomes, allowing results of studies to be compared and combined and for new effective treatments to made available more quickly.

**Trial registration:**

The COS study, of which this is a part, is registered in the Core Outcome Measures in Effectiveness Trials (COMET) database, http://www.comet-initiative.org/studies/details/956. Registered January 2017.

**Electronic supplementary material:**

The online version of this article (10.1186/s13063-018-2805-2) contains supplementary material, which is available to authorized users.

## Background

The global prevalence of diabetes was estimated to be 8.8% of adults 20–79 years in 2017, and by 2045 it is predicted that diabetes will affect 623 million people in this age bracket, a 48% increase from 2017 [1], with type 2 diabetes accounting for the majority of cases [2, 3]. Whilst prevention of type 2 diabetes is a key focus for health care [4], it is inevitable that a proportion of those considered to be at high risk or “pre-diabetic” will go on to develop type 2 diabetes and require intervention to normalise their blood glucose [5, 6].

Type 2 diabetes is characterised by abnormal glucose metabolism brought about by resistance to insulin action and an inadequate compensatory insulin secretory response [7, 8]. Treatment of type 2 diabetes ultimately aims to control glycaemia, by lifestyle changes or pharmacotherapy, to avoid hyperglycaemia and associated long-term complications. Pharmacotherapy for type 2 includes a number of classes of pharmaceutical agents which affect glycaemia through varying cellular mechanisms and resulting physiological actions [9, 10]. Lifestyle changes focus on changes to physical activity and dietary intake with body weight management being the primary focus [11–15]. These methods may also be supplemented with bariatric surgical intervention [16].

The primary and secondary outcomes used in clinical trials of glucose-lowering interventions are varied, and systematic reviews identify inconsistency in the outcomes measured and reported. Although many trials report glycated haemoglobin, other measures of glycaemic control and outcomes relating to hypoglycaemia, mortality, diabetes-related complications and quality of life are less frequently reported [3–8]. In a recent systematic review of open recruiting trials registered with ClinicalTrials.gov, no single outcome was measured in 100% of the included studies [17], and this heterogeneity in outcomes may have an impact on the translatability of trials into benefits for patients. [18, 19].

The relevance and consistency of trial outcomes may be improved by the development of a core outcome set (COS) that represents the minimum set of outcomes that should be measured and reported in any clinical trial for a given condition, in this case type 2 diabetes [13–15].

### Aims and objective

The aim of this study is to contribute to the development of an international COS, relevant to glucose-lowering interventions for use in studies of adults with type 2 diabetes.

The specific study objectives are as follows: to identify a list of outcomes used in current, open clinical trials registered on ClinicalTrials.gov together with outcomes reported in qualitative literature relevant to type 2 diabetes; to prioritise outcomes from the health care professional perspective; to prioritise outcomes from the perspective of patients with type 2 diabetes; to prioritise outcomes from the perspective of researchers in the field and policy makers; and to integrate the outcomes important to all stakeholders into a combined COS.

### Identifying existing knowledge

A search of the Core Outcome Measures in Effectiveness Trials (COMET) initiative database (http://www.comet-initiative.org/) was completed prior to commencing this project (on 21 October 2016). No published or ongoing COS for trials involving patients with type 2 diabetes without co-morbidity was identified.

### Scope of the core outcome set

This COS will be developed for effectiveness trials evaluating any non-surgical therapeutic intervention for the treatment of hyperglycaemia in adults with type 2 diabetes. Surgical interventions will not be included, as these outcomes are captured in an existing COS for bariatric and metabolic surgery [20]. Type 1 diabetes, gestational diabetes, type 2 diabetes in children and maturity-onset diabetes of the young (MODY) are outside the scope of this COS.

### Study oversight

An international Study Steering Committee (SSC) comprising three health care professionals, four public contributors and a policy maker with experience in COSs has been established. The remit of the SSC is to oversee the SCORE-IT study, provide feedback on the study protocol and list of outcomes, contribute to the dissemination of the online Delphi survey and to contribute to the final consensus meeting and dissemination of the COS.

## Methods/design

**Fig. 1:**
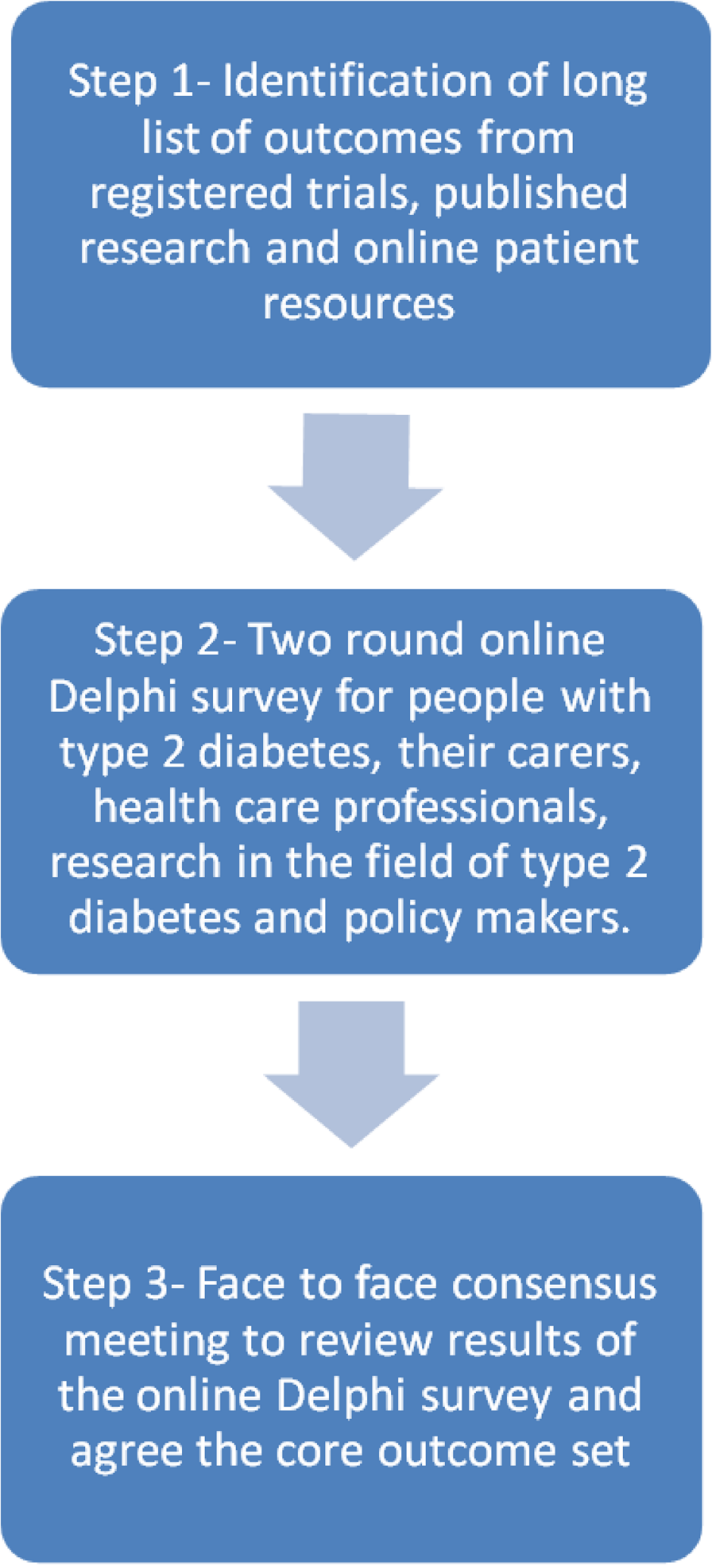
Overview of COS development process

The COS development process will involve the steps shown in Fig. 1. Each of these is described below.

### Step 1: identification of outcomes

The list of outcomes for use in an online Delphi survey will be generated from the following sources:

1. A systematic review of open and recruiting trials registered with ClinicalTrials.gov. The search strategy and results of this systematic review have been published elsewhere [17].
2. Identification of additional outcomes from long-term cardiovascular outcome studies. Guidance released by the Food and Drug Administration (FDA) required clinical trials for the treatment of type 2 diabetes to demonstrate cardiovascular safety of the new treatment by including specific cardiovascular outcomes [21]. The outcomes described by the FDA will be reviewed against the outcomes identified from open, registered trials, and new outcomes will be included in the initial list of outcomes for round 1 of the Delphi. Trials initiated in response to the FDA guidance were long-term follow-up studies that have now been completed or are nearing completion. As most of these studies are no longer recruiting, they will not be identified in the ClinicalTrials.govsearch results. Instead a list of the key trials that opened between 2008 and 2016 will be provided by the SSC and outcomes extracted.
3. Health Talk Online is a public website containing information and patient interviews for a range of health conditions. The experiences of patients with type 2 diabetes shared as video clips on Health Talk Online will be reviewed and potential outcomes extracted [22]. Only transcripts of patients aged 18 and older without a co-morbidity will be used.
4. Outcomes will be identified from a narrative synthesis of the qualitative literature. The protocol for the narrative synthesis and resulting outcomes has been submitted for publication separately. Briefly, a search of the literature in MEDLINE, with no restrictions on date, will be undertaken. The search terms, described in Table 1, comprise empirically tested qualitative methodological filters designed to identify qualitative research from the MEDLINE electronic database with maximum specificity [23]. Abstracts will be screened, and the full text will be reviewed for articles meeting the following inclusion criteria: participants are patients with type 2 diabetes; the focus is type 2 diabetes and not an associated co-morbidity; and qualitative methods (interviews and/or focus groups) have been used.
5. A recent study by Reaney et al. has examined patient-reported outcome measures (PROMs) used in phase 3 clinical trials of glucagon-like peptide 1 (GLP-1) receptor agonists, novel insulins, sodium-glucose co-transporter 2 (SGLT-2) inhibitors and dipeptidyl peptidase-4 DPP-4 inhibitors [24]. The outcome domains in these PROMS will be reviewed against those identified in sources 1–4 for additional outcomes.
6. The Cochrane Database of Systematic Reviews will be searched for “type 2 diabetes” in the title, abstract and keywords. Eligible reviews will be those of glucose-lowering interventions for type 2 diabetes. The outcomes used in the included Cochrane reviews will be extracted and compared against the list of outcomes identified in sources 1–4 for additional outcomes.
7. The EUropean Best Information through Regional Outcomes in Diabetes (EUBIROD) project’s BIRO common data set for clinical practice will be reviewed for potentially relevant outcomes that have not been identified from the systematic review or other sources [25].

**Table 1:** MEDLINE search strategy

Multi-field search		
	(type 2 diabetes OR type II diabetes)	Abstract
AND	patient*	Abstract
AND	(Qualitative OR Themes)	Abstract
AND	(symptom OR treatment OR living with)	Abstract
NOT	(co-morbid* OR foot ulcer* OR retinopathy OR nephropathy OR bariatric surgery OR non-alcoholic fatty liver disease OR cardiovascular disease)	Abstract

#### Review of the final outcome list

The list of outcomes from all sources will be reviewed by NLH and JW to group like outcomes together and categorise each outcome according to a 38-item taxonomy [26]. The outcomes list will then be reviewed by members of the SSC to ensure that outcomes have been grouped appropriately, the outcome description is clear and that there are no duplications within the outcomes. The SSC will also take into consideration the number of outcomes and may request that outcomes be further condensed and combined or that outcomes be removed if measured in a single or small number of studies or if they are deemed to be irrelevant to glucose-lowering interventions. This approach will ensure that the number of outcomes to be scored by participants of the online Delphi survey is manageable.

### Step 2: prioritisation of outcomes

#### Stakeholder involvement

Stakeholder groups representing health care professionals, people with type 2 diabetes, researchers in the field and policy makers will be invited to participate in the consensus process (two-round online Delphi survey and face-to-face consensus meeting) (Table 2).

**Table 2:** Summary of stakeholders

Key stakeholder group	Stakeholder grouping for presentation of round 1 results in round 2 of the Delphi survey	Question participants asked to consider in the online Delphi
People with type 2 diabetes	People with type 2 diabetes and their carers	“What sorts of changes in your diabetes management, symptoms or your day-to-day life would tell you that a treatment was actually helping you and what would be important in helping you decide if it’s not?”
Carers of someone with type 2 diabetes		
Health care professionals treating people with type 2 diabetes: Consultants Specialist nurses Dietitians Pharmacists General practitioners Clinical psychologists Physical therapists Exercise specialists	Health care professionals	“When treating patients with type 2 diabetes, what results of treatment do you consider to be the most important?”
Researchers in the field of type 2 diabetes	Researchers	“When treating patients with type 2 diabetes, what results of treatment do you consider to be the most important?”
Health care policy makers (Health Technology Assessment representatives)	Health care policy makers	

People with type 2 diabetes may be older with limited access to an online survey. At the time of registration participants will be asked which 10-year age bracket they fall into. The number of participants in each age range will be monitored to ensure representativeness of the population.

We propose to contact potential participants via national and international professional bodies and patient organisations; these may include but are not limited to those described in Additional file 1.

### Step 3: online Delphi survey

A list of outcomes identified from the sources described in step 1 will be prioritised in a two-round online Delphi survey. The Delphi method allows anonymous review and scoring of outcomes in a way that gives equal influence to all who participate, avoids an individual participant being overtly influenced by the opinions of any other participant, facilitates international contribution and, as there is no direct contact between participants, provides a mechanism for reconciling different opinions [27–29].

Outcomes will be categorised using the outcomes taxonomy developed by Dodd et al. [26]. The outcomes list will be presented grouped into the core areas of death, physiological or clinical, life impact, resource use or adverse events. The list will then be ordered within these domains according to the 38 categories within the taxonomy.

Participants of the Delphi will be asked to score each outcome using the Grading of Recommendations Assessment, Development and Evaluation (GRADE) nine-point Likert scale [30]. In the Delphi process the scale will be presented in the format 1 to 9, with 1 to 3 labelled “not important”, 4 to 6 labelled “important but not critical” and 7 to 9 labelled “critical”. An option of “unable to score” will also be included together with an option to add a comment to each outcome. All outcomes will be written in plain language and the descriptions used reviewed and contributed to by members of the SSC, including public contributors, and the same descriptions used for all stakeholders. Participants may also add any additional outcomes they think important but not already included on the list. Any additional outcomes suggested will be reviewed by two members of the study team and new outcomes agreed on by the SSC.

In the second round of the online Delphi survey, responses for each stakeholder group will be summarised for each outcome and displayed graphically as the percentage of each stakeholder group who has given each score. All outcomes scored in round 1 will be carried forward to round 2. Participants will be able to view the grouped responses together with their own score in round 1 and will be asked to re-score the outcome based on this information using the same 1–9 scale. Participants may choose to change their score or to keep it the same. New outcomes identified from free text responses in round 1 will be presented in round 2 alongside the verbatim text that led to the outcome. Participants will be asked to score these new outcomes without reflection. Round 2 responses will be analysed using descriptive statistics and summarised according to a predefined definition of consensus (Table 3).

**Table 3:** Definition of consensus

Consensus classification	Description	Definition
Consensus in	Consensus that outcome should be included in the core outcome set	70% or more participants scoring as 7–9 AND < 15% participants scoring as 1–3
Consensus out	Consensus that outcome should not be included in the core outcomes set	50% or fewer participants scoring 7–9 in each stakeholder group.
No consensus	Uncertainty about importance of outcome	Anything else

All rounds of the Delphi will be delivered online using bespoke web-based Delphi software (DelphiManager) designed, hosted and delivered by the University of Liverpool. Registration for participation in the survey will include specific consent questions. Completion of the survey will then be considered to imply informed consent.

### Step 4: consensus meeting

The final step in the consensus process will be a face-to-face consensus meeting to discuss the results of the online Delphi survey and ratify the COS. The meeting will be chaired by an independent facilitator. Participants of the Delphi who have completing both rounds and who express an interest in attending will be randomly selected to attend to ensure that there are similar numbers from each stakeholder group. Results of the two-round Delphi will be presented as outcomes meeting the definition of consensus (Table 3) [27]. Where outcomes have reached consensus in or consensus out during the Delphi, participants will have the opportunity to voice opinion should they disagree with inclusion/exclusion of the outcome in the COS. Participants raising an objection will be invited to provide further information before all participants of the consensus meeting to re-score the outcome. Where outcomes have not reached consensus during the Delphi, they will be discussed and participants of the consensus meeting invited to re-score the outcome.

### Ethical considerations

The COS process will generate generalisable findings that can be extrapolated from the present study to a broader population, and therefore ethical approval has been sought from the University of Liverpool Ethics Committee prior to undertaking the consensus methods (online Delphi and consensus meeting) ref.: 3306.

## Discussion

There is currently no published COS for type 2 diabetes. The development of a COS in this clinical area aims to improve the interpretation and comparison of future studies and reduce the risk of outcome reporting bias and heterogeneity across studies. The SCORE-IT study will involve multiple key stakeholder groups to ensure that the COS is suitable and well accepted in future research.

### Study status

The SCORE-IT study is ongoing with the consensus meeting expected to be completed in December 2018.

## Additional file

Additional file 1: Summary of organisations that will be approached to identify participants. (DOCX 15 kb)

## Abbreviations

COMET: Core Outcome Measures in Effectiveness Trials
COS: Core outcome set
DPP-4: Dipeptidyl peptidase-4
FDA: Food and Drug Administration
GLP-1: Glucagon-like peptide 1
GRADE: Grading of Recommendations Assessment, Development and Evaluation
ICHOM: International Consortium for Health Outcomes Measurement
MODY: Maturity-onset diabetes of the young
SGLT 2: Sodium-glucose co-transporter 2
SSC: Study Steering Committee
