# Supplementary material for: Selecting Core Outcomes for Randomised Effectiveness trials In Type 2 Diabetes (SCORE-IT): study protocol for the development of a core outcome set
Source: Trials. 2018 Aug 7;19:427. doi: 10.1186/s13063-018-2805-2 (PMC6081933; doi:10.1186/s13063-018-2805-2)
Supplement: Supplementary file 1 — Summary of organisations that will be approached to identify participants. (DOCX 15 kb) [file 13063_2018_2805_MOESM1_ESM.docx]

| **Additional file 1. Summary of organisations that will be approached to identify participants** | | | |
| --- | --- | --- | --- |
| **Organisation name** | **Coverage** | **Stakeholders** | **Mode of contact requested** |
| Diabetes UK | UK | Patients and health care professionals | Email to registered members  Link on main website  Link via Facebook page  Link on twitter  e-newsletter |
| European Association for the Study of Diabetes | Europe | Health Care Professionals | Email to registered members  Link on main website  Link via Facebook page  Link on twitter |
| International Diabetes Federation | Africa  Europe  Middle East and North Africa  North America and Caribbean  South and Central America  South East Asia  Western Pacific | Patients and health care professionals | Email to registered members  Link on Facebook page  Link on twitter |
| American Diabetes Association | North America | Health care professionals  Patients | Email to members  Online community for patients  (<http://community.diabetes.org/home>)  Link on Facebook page  Link on twitter  Diabetes Forecast Magazine (published monthly online and in print <http://www.diabetesforecast.org/about-forecast/editorial-contacts.html>) |
| Diabetes Canada | Canada | Health Care professionals  patients | Monthly e-newsletter  Email to professional members  Email to patient members  Link on Facebook page  Link on twitter |
| British Dietetic Association | UK | Health Care Professionals | Email to members (specialist group for diabetes)  <http://www.diabetesdietitians.org.uk/contact/>  Link on Facebook page  Link on twitter |
| The British Psychological Society | UK | Health Care Professionals | Email to members.  Link on Facebook page  Link on twitter |
| International Union of Nutritional Sciences | Europe  Middle East and North Africa  North America and Caribbean  South and Central America  South East Asia  Western Pacific | Researchers an health care professionals | Email to each of the adhering bodies registered with the IUNS http://www.iuns.org/adhering-bodies/ |
